# Genetic bad cholesterol drops 60% in Australian drug trial

**Published:** 2023

Australian researchers have found a potential oral medication developed to lower, by as much as 65%, levels of ‘bad’ cholesterol – lipoprotein(a) or Lp(a) – which affects some 20 to 25% of people around the world. Until now, there has been no cure or approved specific treatment for lowering these levels, reports Medical News Today, and because Lp(a) levels are genetically inherited, lifestyle changes such as diet and exercise that may benefit other types of cholesterol do not help.

Now, researchers from Monash University’s Victorian Heart Institute and Victorian Heart Hospital have found that an experimental oral medication developed to target Lp(a) was able to lower its levels by more than half during a firstin- human phase one clinical trial. This study was recently published in the journal J Am Med Assoc.

## What is lipoprotein(a)?

Lipoproteins are a type of protein that transports cholesterol through the blood. There are two main types of lipoproteins: high-density lipoprotein (HDL) cholesterol, considered ‘good’, and low-density lipoprotein (LDL) cholesterol, considered ‘bad’.

Although the body needs some cholesterol for certain functions, too much LDL cholesterol can lead to atherosclerosis – where cholesterol builds up to form plaques on the inside walls of arteries, making it hard for blood to pump through.

Lp(a) is a form of LDL cholesterol that is ‘stickier’ than other types, making it easier for the build-up to occur and arteries to become blocked. The amount of Lp(a) in a person’s system is determined by their genetic history and ethnicity. For example, African Americans are at an increased risk for high Lp(a) levels compared with other ethnic groups. Having a high level of Lp(a) can increase the risk of cardiovascular diseases such as coronary heart disease and stroke.

## Muvalaplin: a weapon against bad cholesterol?

In this study, researchers conducted a clinical trial to assess an experimental medication called muvalaplin. ‘Genetic and population studies show us that high Lp(a) levels are associated with a high risk of heart disease,’ Dr Stephen Nicholls, cardiologist and director of Monash University’s Victorian Heart Institute and the Victorian Heart Hospital at Monash Health and lead author of this study, told Medical News Today.

‘As much as 20% of the population have high levels. We don’t currently have specific therapies that lower levels, which may be important in the prevention of heart disease.’ He and his team looked at how well the drug worked, as well as its safety and tolerability in humans.

‘Lp(a) forms when an LDL particle binds to the protein Apo(a). Muvalaplin essentially blocks that binding from happening in the liver and therefore prevents the formation of Lp(a). It would provide an oral option for the treatment of patients with high Lp(a) levels to reduce their risk of heart disease.’

## Muvalaplin lowers Lp(a) by up to 65%

For this phase one clinical trial, the team recruited 114 healthy participants of different genders and ethnic backgrounds. The purpose was to assess the safety and tolerability of muvalaplin, its pharmacokinetics, as well as indicators of the drug’s effect on the target, Lp(a). Participants either received a single dose of muvalaplin, an ascending dose where the amount taken was increased over time, or a placebo for 14 days.

At the end of the study, the Lp(a) levels of those who had received muvalaplin daily over the 14 days had dropped by as much as 65%. The drug was not associated with any tolerability concerns or clinically significant adverse effects. The most commonly reported side effects included headache, back pain, fatigue, abdominal pain, diarrhoea and nausea.

## Under-recognised heart disease risk factor

Dr Cheng-Han Chen, an interventional cardiologist and medical director of the Structural Heart Programme at MemorialCare Saddleback Medical Centre in California, not involved in the research, said this research was definitely a step in the right direction. ‘Lp(a) is a very hot topic right now in heart disease,’ he said. ‘A number of studies are investigating how we can improve a patient’s health outcomes by covering that drug.

‘There (are) other agents that are in clinical trials right now but they’re all injections… a patient would rather get pills than injections. It’s a big step in the right direction in terms of getting people be able to just take a pill rather than an injection.’

## How else to lower Lp(a) levels

Right now, the only Food and Drug Administration-approved therapy for lowering Lp(a) levels is lipoprotein apheresis. This process physically removes lipoproteins from the blood and is only available for people with certain Lp(a) levels and other risk factors. Researchers are currently looking at PCSK9 inhibitors as a possible treatment and a number of drug candidates are undergoing clinical trials.
